# Death receptor 6 is a novel plasmacytoid dendritic cell-specific receptor and modulates type I interferon production

**DOI:** 10.1007/s13238-015-0239-0

**Published:** 2016-02-24

**Authors:** Jingyun Li, Qiumei Du, Rui Hu, Yanbing Wang, Xiangyun Yin, Haisheng Yu, Peishuang Du, Joël Plumas, Laurence Chaperot, Yong-jun Liu, Liguo Zhang

**Affiliations:** Key Laboratory of Immunity and Infection, Institute of Biophysics, Chinese Academy of Sciences, Beijing, 100101 China; University of Chinese Academy of Sciences, Beijing, 100049 China; Department of Research and Development, Etablissement Français du Sang Rhône-Alpes Grenoble, 38701 La Tronche, France; Baylor Institute for Immunology Research, Baylor Research Institute, Dallas, TX 75204 USA

**Dear Editor,**

Plasmacytoid dendritic cells (pDCs) are the professional type I interferon-producing cells of the immune system, which rapidly produce massive amounts of type I interferons (IFN-I) in response to viruses or other nucleic acids ligands through selectively expressed toll-like receptor (TLR)-7 and TLR9 (Siegal et al., 1999). The activation of pDCs not only inhibits virus replication, but also regulates the function of other immune cells and links the innate and adaptive immunity (Liu, 2005). The local accumulation of pDCs has been reported in both suppressive and overactive immune status (Swiecki and Colonna, 2010), which highlights the importance of characterizing the molecular mechanisms underlying the functional specialization of pDCs in IFN-I production.

Death receptor 6 (DR6) is a member of death receptor family, which belongs to the tumor necrosis factor receptor superfamily (TNFRSF). It is reported that DR6 plays vital roles in axon pruning, neuron death, and negatively regulates oligodendrocyte survival, maturation and myelination in neural system (Nikolaev et al., 2009; Mi et al., 2011). DR6^-/-^ mice exhibit enhanced CD4^+^ T cell proliferation, Th2 cytokines production and B cell expansion, survival, and humoral responses, which imply that DR6 plays important roles in murine immune responses (Liu et al., 2001; Schmidt et al., 2003). However, the expression profiles and functions of DR6 in human immune system remain largely unknown.

We found that DR6 was highly expressed in human pDCs comparing with other blood cells by microarray analysis (Fig. 1A). Additionally, we found that among the 6 death receptors, including TNFR1, CD95, DR3, DR4, DR5 and DR6, pDCs exclusively expressed DR6 but not others (Fig. 1A). To confirm the expression of DR6 in pDCs, we performed quantitative real time-PCR analysis on several cell types from peripheral blood. Consistently, DR6 mRNA was preferentially expressed on human pDCs (Fig. 1B).

**Figure 1:**
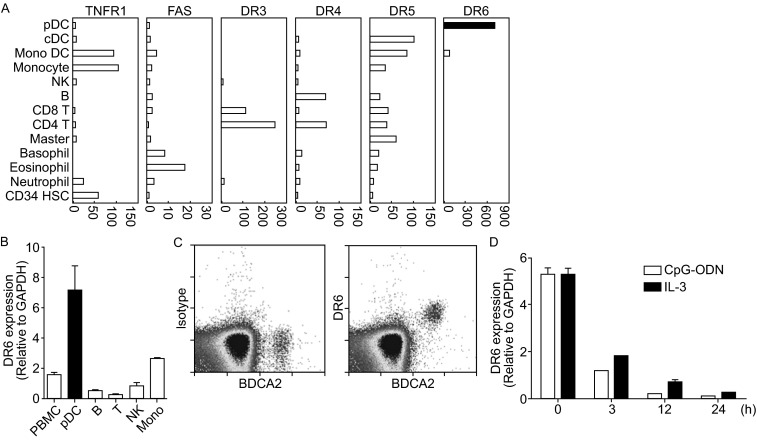
**DR6 is specifically expressed on human pDCs**. (A) The relative expression of death receptors in different subsets of peripheral blood leukocytes was compared by cDNA array. (B) Human total PBMC, pDCs, B cells, T cells, NK cells, monocytes were isolated and total RNA was purified and reverse transcribed. The cDNA was subjected to quantitative real-time PCR analysis and the arbitrary units of gene expression were normalized with GAPDH. Results are the mean value of duplicates, and error bars represent standard deviations (SD). (C) Human PBMCs were incubated with isotype control (left panel) or α-DR6 (right panel) followed by biotin labeled rat anti-mouse IgG and streptavidin-PE, then both samples were stained with BDCA2-APC. (D) pDCs were cultured with CpG-B (1 μmol/L) or IL-3 (20 ng/mL) for 3~24 h and the cells were harvested and the levels of DR6 RNA were evaluated by quantitative real-time PCR

To verify the expression of DR6 on pDCs at the protein level, we used αDR6, homemade DQM3 (Hu et al., 2014), to stain fresh human PBMC. BDCA2 was used to identify pDCs (Dzionek et al., 2000). Our results showed that almost all DR6 positive cells were BDCA2 high cells, suggesting that DR6 is indeed specifically expressed on pDCs at protein level (Fig. 1C). In addition, we also demonstrated that pDCs rapidly down-regulated the expression of DR6 upon *in vitro* culture with CpG-ODN or IL-3 as early as 3 h after activation (Figs. 1D and S1A). GEN2.2 is a human leukemic cell line and similar to human primary pDCs both phenotypically and functionally (Chaperot et al., 2004). Our Data showed that DR6 was also downregulated by CpG-ODN stimulation in GEN2.2 cells. However, there was no obvious DR6 downregulation when GEN2.2 cells were cultured for up to 24 h in medium in the absence of stimulation (Fig. S1B). Therefore, our results point out that DR6 is a novel marker of pDCs and rapidly downregulated upon activation.

To analyze the function of DR6 in human pDCs, we performed knockdown experiments in GEN2.2 cells. We constructed lentiviral vectors expressing two short hairpin RNAs (shRNA-1 and shRNA-2) specific for DR6 and established stably transduced GEN2.2 cell lines. The knockdown efficiency was confirmed by quantitative real time-PCR (Fig. 2A). DR6 knockdown cells (shRNA-1 or shRNA-2) and control cells (shRNA-c) were treated with CpG-ODN and the secretion of IFN-α and IL-6 was examined by ELISA. Knockdown of DR6 by shRNA-1 or shRNA-2 significantly reduced IFN-α production in response to CpG-ODN (Figs. 2B, 2D, S2A and S2B), while it had marginal effect on IL-6 production (Fig. 2C and 2E). Besides, our data indicated that knockdown DR6 also significantly reduced CpG-B induced IFN-β production (Fig. S2C). These findings suggest that DR6 plays an important role in IFN-I production, but not in the production of proinflammatory cytokines.

**Figure 2:**
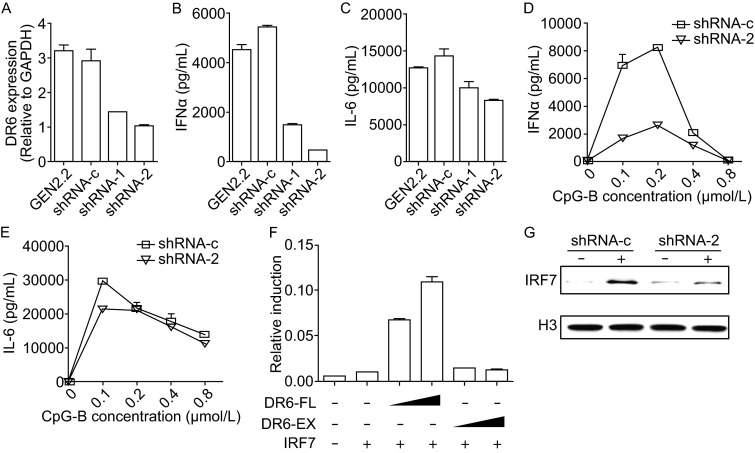
**DR6 regulates type I IFN-production by mediating CpG-ODN induced IRF7 nuclear translocation**. (A) GEN2.2 cells were transduced with lentiviral vectors carrying either scrambled shRNA (shRNA-c) or shRNA targeting DR6 (shRNA-1 or shRNA-2) and stable cell lines were generated. The knockdown efficiency was confirmed by quantitative real-time PCR. (B–E) DR6 knockdown cells and control cells were stimulated with CpG-B for 20 h. Levels of IFN-α and IL-6 in the culture supernatants were examined by ELISA. (F) HEK293T cells were transiently transfected with IRF7 and IFN-α4 promoter-driven luciferase reporter plasmid together with increasing amounts of expression vectors of DR6 (DR6-FL) or truncated DR6 without intracellular domain (DR6-EX). Renilla was used as an internal control for transfection efficiency. Promoter activity was measured 24 h post transfection by luciferase assay. (G) DR6 knockdown cells (shRNA-2) and control cells (shRNA-c) were stimulated with CpG-B for 4 h. Nuclear fractions were isolated and immunoblotted with anti-IRF7 antibody. Histone H3 was used as a loading control

To further investigate the roles of DR6 in regulating interferon responses, we tested the ability of DR6 to activate IFN-α promoter by a reporter assay. HEK293T cells were transiently transfected with the various amounts of expression plasmid encoding full length DR6 (DR6-FL) or intracellular-domain deleted DR6 (DR6-EX) along with IFN-regulatory factor 7 (IRF7) (Kawai et al., 2004) and a reporter plasmid driven by IFN-α4 promoter. Luciferase activity was measured at 24 h post transfection. Overexpression of DR6-FL caused activation of IFN-α4 promoter in a dose-dependent manner, whereas there was little induction of IFN-α4 promoters activity when DR6-EX was overexpressed (Fig. 2F). These data indicate that DR6 regulating IFN-I production depends on its intracellular domain.

It is well established that IRF7 nuclear translocation is the key event upon stimulation pDCs with CpG-ODN, which eventually leads to the production of IFN-I (Honda et al., 2005a; Honda et al., 2005b). To investigate the requirement of DR6 for CpG-ODN induced nuclear translocation of IRF7, we isolated nuclear fractions following CpG-ODN treatments and monitored the nuclear localization of IRF7 by Western blot. After CpG-ODN stimulation, IRF7 displayed increased distribution in the nucleus, while knockdown of DR6 in GEN2.2 cells significantly diminished the nuclear localization of IRF7 (Fig. 2G). This result suggests that DR6 is necessary for CpG-ODN induced IRF7 nuclear translocation.

In summary, in this study, we report for the first time that DR6 is a novel human pDC-specific receptor. In addition, we demonstrated that knockdown DR6 by shRNA in human pDCs cell line GEN2.2 significantly diminished the CpG-ODN induced IRF7 nuclear localization and IFN-I production. Therefore, as a regulator of interferon production by pDCs, DR6 may be a potential targets for regulating IFN-I production by pDCs.

Pan et al. (1998) reported that DR6 overexpression in some cell lines induced NF-κB and JNK activation. As a matter of fact, we found that both NF-κB and JNK activation upon CpG-ODN treatment were inhibited in DR6 knockdown cells (unpublished data). We hypothesize that DR6 might regulate TLR-9 induced IRF-7 translocation and IFN-I production through NF-κB activity and/or JNK activation, while the exact attribution needs further deep investigation.

DR6 belongs to death receptor family which initiates cytotoxic signals or regulate cell proliferation and survival (Guicciardi and Gores, 2009). During our research, the cell growth rates of GEN2.2 did not change upon knocking-down DR6 by shRNA, while DR6 overexpression on GEN2.2 does increase cell death (data not shown). Thus, the roles of DR6 in pDCs development and activation need to be further characterized.

N-terminal of amyloid precursor protein (N-APP) was identified as a DR6 ligand, leading to caspase-3 activation in neurons (Nikolaev et al., 2009). However, DR6 negatively regulates survival and maturation through the caspase-3 pathway by a mechanism independent of N-APP binding in oligodendrocyte (Mi et al., 2011). Recently, it is reported that high-affinity binding to DR6 requires a C-terminal portion of the APP ectodomain rather than its N-terminal domain (Olsen et al., 2014). However, if APP regulates pDCs development and functions through DR6 has not been characterized. Furthermore, it will also be necessary to address if other DR6 ligands exist and regulate DR6 signaling in pDCs in human immune system.

## Electronic supplementary material

Below is the link to the electronic supplementary material.

Supplementary material 1 (PDF 313 kb)
