# Supplementary material for: Death receptor 6 is a novel plasmacytoid dendritic cell-specific receptor and modulates type I interferon production
Source: Protein Cell. 2016 Feb 24;7(4):291–4. doi: 10.1007/s13238-015-0239-0 (PMC4818848; doi:10.1007/s13238-015-0239-0)
Supplement: Supplementary file 1 — Supplementary material 1 (PDF 313 kb) [file 13238_2015_239_MOESM1_ESM.pdf]

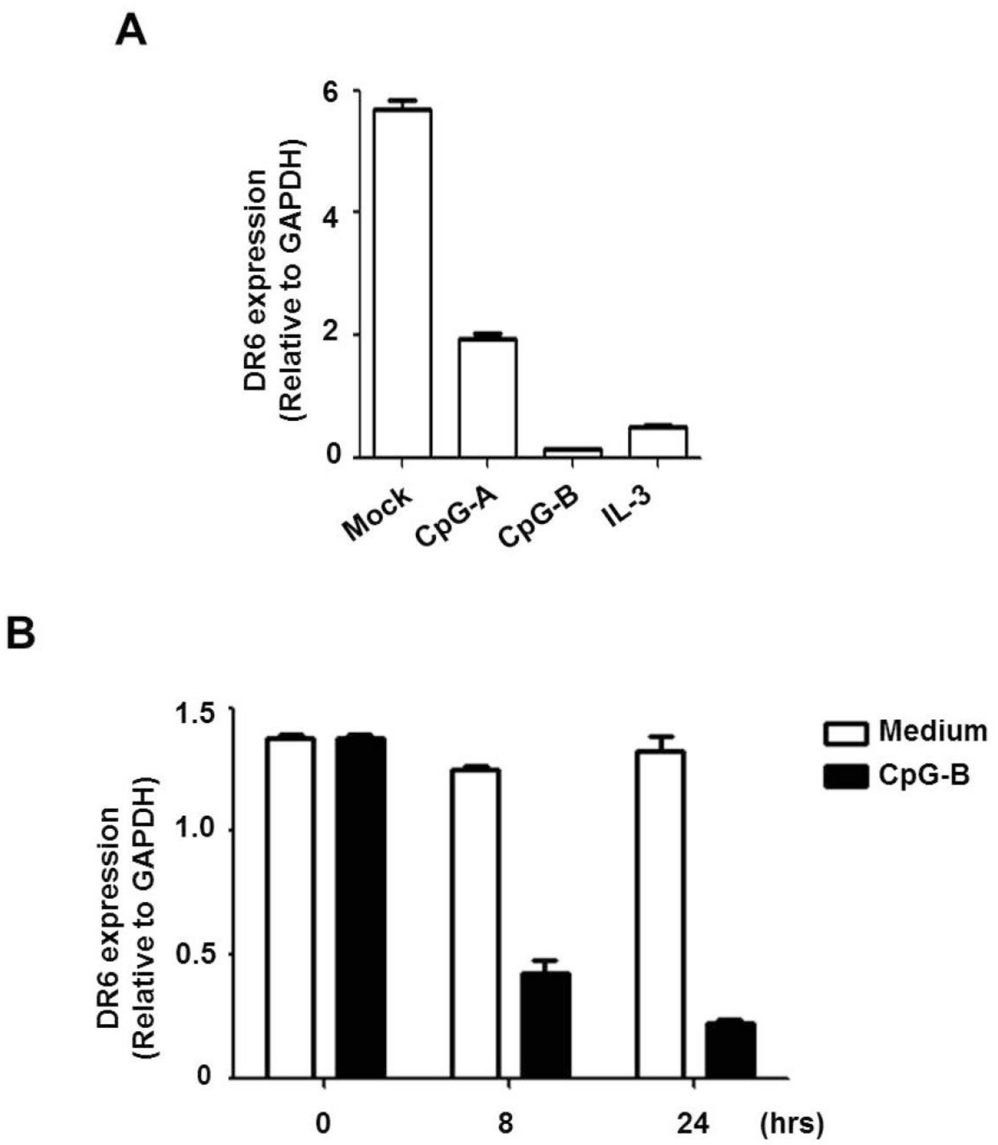

Figure S1. DR6 is downregulated upon activation.

(A) pDCs were harvested as soon as purified (mock) or cultured with CpG-A (1µM) / CpG-B (1µM) / IL-3 (20ng/ml) for 24 hours and then harvested. The levels of DR6 RNA were evaluated by quantitative real-time PCR. (B) GEN2.2 cells were cultured with medium only or stimulated with CpG-B (0.2µM) for 0~24 hours. Cells were harvested and the levels of DR6 RNA were evaluated by quantitative real-time PCR.

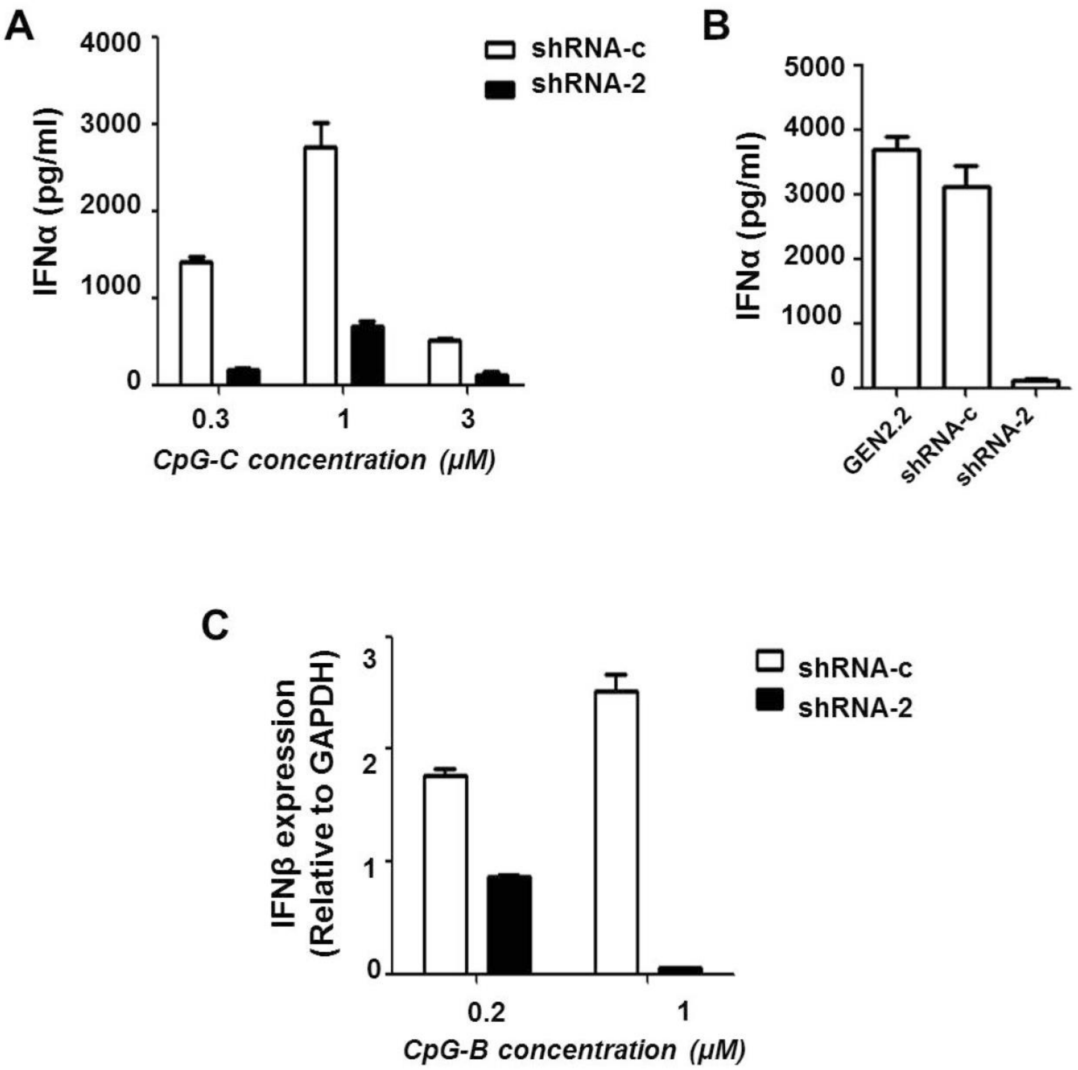

Figure S2. DR6 regulates CpG-ODN induced IFN-I production.

(A) DR6 knockdown cells and control cells were stimulated with CpG-C (0.3μM, 1μM and 3μM) for 20 hours. Levels of IFN-α in the culture supernatants were examined by ELISA. (B) DR6 knockdown cells and control cells were stimulated with CpG-A (1μM) for 20 hours. Levels of IFN-α in the culture supernatants were examined by ELISA. (C) DR6 knockdown cells and control cells were stimulated with CpG-B (0.2μM and 1μM) for 6 hours. Levels of IFN-β RNA were measured by quantitative real-time PCR.
